# New Antimalarial Hits from *Dacryodes edulis* (Burseraceae) - Part I: Isolation, *In Vitro* Activity, *In Silico* “drug-likeness” and Pharmacokinetic Profiles

**DOI:** 10.1371/journal.pone.0079544

**Published:** 2013-11-08

**Authors:** Denis Zofou, Esther Laure Tematio, Fidele Ntie-Kang, Mathieu Tene, Moses N. Ngemenya, Pierre Tane, Vincent P. K. Titanji

**Affiliations:** 1 Biotechnology Unit, Faculty of Science, University of Buea, Buea, Cameroon; 2 Laboratory of Natural Products Chemistry, University of Dschang, Dschang, Cameroon; 3 CEPAMOQ, University of Douala, Douala, Cameroon; 4 Chemical and Bioactivity Information Centre, Faculty of Science, University of Buea, Buea, Cameroon; Ehime University, Japan

## Abstract

The aims of the present study were to identify the compounds responsible for the anti-malarial activity of *Dacryoedes edulis* (Burseraceae) and to investigate their suitability as leads for the treatment of drug resistant malaria. Five compounds were isolated from ethyl acetate and hexane extracts of *D. edulis* stem bark and tested against 3D7 (chloroquine-susceptible) and Dd2 (multidrug-resistant) strains of *Plasmodium falciparum*, using the parasite lactate dehydrogenase method. Cytotoxicity studies were carried out on LLC-MK2 monkey kidney epithelial cell-line. *In silico* analysis was conducted by calculating molecular descriptors using the MOE software running on a Linux workstation. The “drug-likeness” of the isolated compounds was assessed using Lipinski criteria, from computed molecular properties of the geometry optimized structures. Computed descriptors often used to predict absorption, distribution, metabolism, elimination and toxicity (ADMET) were used to assess the pharmacokinetic profiles of the isolated compounds. Antiplasmodial activity was demonstrated for the first time in five major natural products previously identified in *D. edulis*, but not tested against malaria parasites. The most active compound identified was termed DES4. It had IC_50_ values of 0.37 and 0.55 µg/mL, against 3D7 and Dd2 respectively. In addition, this compound was shown to act in synergy with quinine, satisfied all criteria of “Drug-likeness” and showed considerable probability of providing an antimalarial lead. The remaining four compounds also showed antiplasmodial activity, but were less effective than DES4. None of the tested compounds was cytotoxicity against LLC-MK2 cells, suggesting their selective activities on malaria parasites. Based on the high *in vitro* activity, low toxicity and predicted “Drug-likeness” DES4 merits further investigation as a possible drug lead for the treatment of malaria.

## Background

The emergence and spread of resistance to frontline anti-malarials is a real challenge to malaria control, which can be addressed by expanding the arsenal of antimalarial products. Medicinal plants are well known sources of antimalarials [1,2]. Over a thousand plant species are commonly used across Africa for prevention and/or treatment of malaria symptoms, and some of these had been revealed as housing uniquely effective antimalarial. The examples of quinine and artemisinin isolated from *Cinchona* sp. and *Artemisia annua* are highly illustrative [2]. 

*Dacryodes edulis* (G Don) also known as *Pachylobusedulis* (G. Don), *Canarium edule* (G. Don) Hook.; *C. saphu* Engl., *Pachylobus edulis* G. Don or *P. saphu* (Engl.) Engl.; is an evergreen tree attaining a height of 18-40 m in the forest but not exceeding 12 m in plantations. The plant which can be cultivated widely (since it adapts well to differences in the duration of day light, temperature, rainfall, soils and altitude), is a multipurpose plant in African folk medicine. In traditional medicine, different preparations of parts of the plant are used variously in Nigeria and the Democratic Republic of Congo to treat several diseases including parasitic skin diseases, jigger, mouthwash, tonsillitis, sickle cells disease, arthritis, wounds and malaria [3–6]. It is taken in a powdered form with *maleguetta* pepper (*Aframomum melegueta*) as an anti-dysenteric, for anaemia and oral bleeding. With palm oil, it is applied topically to relieve general pains and stiffness, and to treat skin diseases. A decoction of the root bark is drunk for leprosy [3]. Leaf sap is instilled into the ear for ear problems, and a leaf decoction is used to prepare a vapour bath for fevers and headache [3]. In the West region of Cameroon, where this plant is locally called *Zo*’o (Batcham), the leaves and the stem bark of *D. edulis* are boiled with leaves of *Cymbopogon citratus* and *Mangifera indica* in water to give a decoction against malaria. In spite of its rich ethnopharmacology, there is data on its antiplasmodial activity.

Previous investigations demonstrated the analgesic, anti-inflammatory, anti-allergic, anti-cancer and antimicrobial and antimalarial activity of *D. edulis* [7-11], and significant antiplasmodial activity had also been recorded for this plant, with IC_50_ below 10 µg/mL on drug resistant malaria parasites [7]. However the bioactive ingredients were yet to be identified. Moreover, the stem bark which is preferably employed in Cameroonian folk medicine is still to be fully investigated. 

Encouraged by the results obtained from the primary screening of extracts from this plant species, the present study was undertaken with the following aims: to isolate, characterize and analyse pure compounds from the stem bark of *D. edulis*, with emphasis on their *in vitro* activities against drug resistant *P. falcipaum* as well as their computer-based “drug-likeness” profiles. 

## Materials and Methods

### Plant collection

The stem bark *D. edulis* was collected in the Batcham village (Bamboutos Division, West Region, Cameroon). *Dacryodes edulis* is widely cultivated in Cameroon for its food use. The plant collection was carried out on a private land, following the permission by the owner (Mr. Mathieu Tezekwe, resident of Balena quarter, Batcham), to conduct the study on this site. The plant species was identified by the Cameroon National Herbarium in Yaoundé, where a voucher specimen (Number 18258/HNC) were deposited. 

### Preparation of crude extracts

The sample was then air-dried in the shade, and powdered. The powder (7 Kg) was macerated in methylene chloride/methanol (1:1) at room temperature for 72 hours, and the filtrate concentrated to dryness using Rotavapor, to a viscous residue stored at 4 °C. 

### Fractionation of extracts and isolation of bioactive compounds

To optimize the isolation of constituents, the dried extract was dissolved in 80% aqueous methanol then subjected to liquid-liquid partition sequentially with hexane, ethyl acetate, and *n*-butanol to separate constituents of varying polarity. The fractionation, purification and characterization of isolated compounds were done as previously described by Tane et al. [12,13] with some modifications [14]. Only extracts or fractions with significant antiplasmodial activity (IC_50_<10 μg/mL) and low or no cytotoxicity (CC_50_>30 μg/mL on Monkey kidney epithelial LLC-MK2 cell-line) were considered for further investigation. Fractionation was performed as described with some modification [15]. Briefly, 100 g of the hexane extract was subjected to silica column chromatography, eluted with *n*-hexane/Acetone gradient, to yield several fractions that were further grouped into 8 main fractions according to their TLC profiles. These fractions were then purified using silica gel column chromatography yielding several mixtures of inseparable compounds and one pure crystalline product codified DES5 after re-crystallization of generated sub-fractions the same product codified DES5. Seventy (70 g) of EtOAc extract was similarly processed eluting with a petroleum ether/EtOAc gradient. This yielded four products codified as follows: DES1, DES2, DES3 and DES4.

The structures of isolated products were elucidated using spectroscopic analysis as previously described [12]. The analyses included, melting point, optical rotation, UV, IR, proton- and carbon 13-NMR (Bruker instrument : 400 or 500 MHz), and mass spectra (GC-MS) were determined.

### *Plasmodium falciparum* culture and maintenance

#### Parasite strains

The 3D7 (MRA-102), and Dd2 (MRA-615) strains were kindly donated by BEI-Resources (MR4, Manassas, VA, USA), and maintained in continuous culture, with back up stored in liquid nitrogen.

#### Parasite culture

The laboratory strains of *P. falciparum* were grown and maintained in culture using the method of Trager and Jensen with some modifications [15,16]. All the chemicals except Albumax II (Gibco; Invitrogen, USA), were ordered from Sigma-Aldrich Inc (Germany). The cultures were monitored and parasitemia assessed using both fluorescence (acridine orange) and normal light (Giemsa stain) microscopes. 

#### Determination of anti-plasmodial activity

The antiplasmodial screen was carried out in 96-well microtitration plates as described by Desjardins et al. [17] with some modifications [14]. The different drug stock solutions were prepared by predisolving the powder in DMSO (200 µL for a final volume of 10 mL stock solution) and subsequently with the culture medium. The parasitaemia was measured using the parasite lactate dehydrogenase (pLDH) assay as previously described [16]. 

As a preliminary study towards the understanding of the mechanisms of action of the active drugs, the stage-specific activity of the products was studied by investigation of the variation in IC_50_ values with exposure time. To achieve this, a sorbitol-synchronized culture with ring stage parasite was exposed to different drugs and the IC_50_s calculated after 24 and 48 hours. 

The drug-interaction patterns of the most active compounds were assessed as earlier described [18-21]. From the results obtained with individual drugs, a stock solution of 32 x IC_50_ of each drug was prepared and used in preparing a series of six combinations (1–6) containing 5:0, 4:1, 3:2, 2:3, 1:4 and 0:5 proportions volume:volume of Drug A versus Drug B respectively. Each of these formulations were then tested and their different inhibitory concentrations determined separately. 

IC_50_ values obtained for the different drug mixtures were used to calculate the Fractional Inhibitory Concentration (FIC) for each drug as described previously. FIC-A for example measures how much the presence of Drug B affects the activity of Drug A in the mixture, and vis versa. FIC>1 is an indication of reduction in activity as a result of the presence of Drug B, FIC=1 shows that there was no effect of Drug B on Drug A, and the FIC<1 reveals an increase in the activity of drug concerned by Drug B. Similarly the effect of Drug A on Drug B is analyzed using FIC-B. 

$$FIC-A=\frac{IC50-A(comb.)}{IC50-A(alone)}$$

$$FIC-B=\frac{IC50-B(comb.)}{IC50-B(alone)}$$

Where FIC-A was the Fractional Inhibitory Concentration of Drug A; IC50-A(comb.), the 50% inhibitory concentration of Drug A when used in combination with Drug B, and IC50-A(alone), the 50% inhibitory concentration of Drug A when tested alone.

Where FIC-B was the Fractional Inhibitory Concentration of Drug B; IC50-B(comb.), the 50% inhibitory concentration of Drug B when used in combination with Drug A, and IC50-B(alone), the 50% inhibitory concentration of Drug B when tested alone.

The activity correlations between Drug A and Drug B were analyzed by non parametric correlation analysis (Spearman) using SPSS Statistics 17.0 (Chicago, USA). Statistical significance was defined as p<0.05.

A graph was constructed with the axes representing the mean FIC of linear scales, with mean FIC-A on the x axis and FIC-B on the y axis. When the combination is additive, the isobole which is the line joining the points that represent all the (x, y) points is straight. Synergistic combinations give concave isoboles, and antagonistic combinations give convex isoboles [20]. The sum of the fractional inhibitory concentrations (ΣFICs) also termed Combination Index (CI) for a particular combination shows the interaction pattern between the two drugs: 

CI=∑FIC=FIC−A+G+FIC−B

CI values were therefore calculated separately for both the drugs present in combinations 2 through 5; combinations 1 and 6 containing Drug A and Drug B alone. In addition to analyzing the result obtained with each drug combination formula, an overall mean CI value for the four combinations was determined to conclude on the interaction patterns of the two drugs being tested. Synergy or antagonism was defined as a mean CI< or >1, respectively, and lack of interaction (also termed additivity) defined as CI= 1 [21].

#### Cytotoxicity study of active compounds

The cytotoxicity of the extracts and pure compounds were estimated on LLC-MK2 monkey kidney epithelial cells as previously described [22] with some modifications [14]. The cell line was ordered from the American Type Culture Collection (ATCC, Manassas, Virginia, USA) and maintained in continuous culture.

### Statistical analysis

#### Analysis of data obtained from pLDH assay

Following the pLDH assay, the optical densities (ODs) of the microtiter plate wells were read and using the software HN-NonLin V1.1 [23] to generate log dose-response correlation coefficients and the 50%, 90%, 95% and 99% Inhibitory Concentrations (IC_50_, IC_90_, IC_95_ and IC_99_ respectively) for the individual replicate tests [14,23]. Each product was tested in triplicate, in two to three separate experiments, giving a total of six to nine repeats per product and per concentration tested. The IC_50_ and CC_50_ values obtained from the replicates were pooled and expressed as geometric mean IC_50_ and standard deviation. The different means were compared among themselves by independent samples t-test. 

#### Analysis of the results of the drug-interaction study

IC_50_ values were used to calculate the Fractional Inhibitory Concentration (FIC) for each drug as described previously [19-21]. FIC-A measures how much the presence of a Drug B affects the activity of Drug-A in the mixture. FIC > 1 is an indication of reduction in activity as a result of the presence of Drug B, FIC = 1 shows that there was no effect of Drug B on A, and the FIC < 1 reveals an increase in the activity of drug concerned by Drug B. Similarly the effect of Drug A on Drug B is analysed using FIC-B. The activity correlations between Drug A and Drug B were analysed by non-parametric correlation analysis (Spearman) using SPSS Statistics 17.0 (Chicago, USA). Statistical significance was defined as p<0.05. A graph was constructed with the axes representing the mean FIC of linear scales, with mean FIC-A on the *x*-axis and FIC-B on the *y*-axis. When the combination is additive, the isobole which is the line joining the points that represent all the (*x*, *y*) points is straight. Synergistic combinations give concave isoboles, and antagonistic combinations give convex isoboles [19].

The sum of the fractional inhibitory concentrations (ΣFICs) also termed Combination Index (CI) for a particular combination shows the interaction pattern between the two drugs. The overall mean CI value for the four combinations formulae was finally determined and synergy or antagonism between the two drugs in combination, defined as a mean CI< or >1, respectively, lack of interaction or additivity defined as CI = 1 [21]. 

#### *In silico* “drug-likeness” analysis and pharmacokinetic prediction

All 3D molecular structures were generated using the graphical user interface (GUI) of the MOE software [24] running on a Linux workstation with a 3.5GHz Intel Core2 Duo processor, and energy minimization was subsequently carried out using the AM1 semiempirical approach implemented in MOPAC_24_ until a gradient of 0.001 kcal/mol was reached. The 3D structures generated were then saved as .mol2 files subsequently included into a MOE database (.mdb). The molar weight (MW), number of rotatable bonds (NRB), lipophilicity parameter [log P_(o/w)_], number of hydrogen bond acceptors (HBA), number of hydrogen bond donors (HBD), total polar surface area (TPSA) and Lipinski violations were calculated using the molecular descriptor calculator included in the QuSAR module of the MOE package [24]. Pharmacokinetic prediction was carried out using 24 essential descriptors related to the absorption, distribution, metabolism, elimination and toxicity (ADMET) of drugs, computed using the QikProp software distributed by Schrodinger Inc. [24-26]. 

## Results

### Characteristics of the isolated compounds

**Figure 1:**
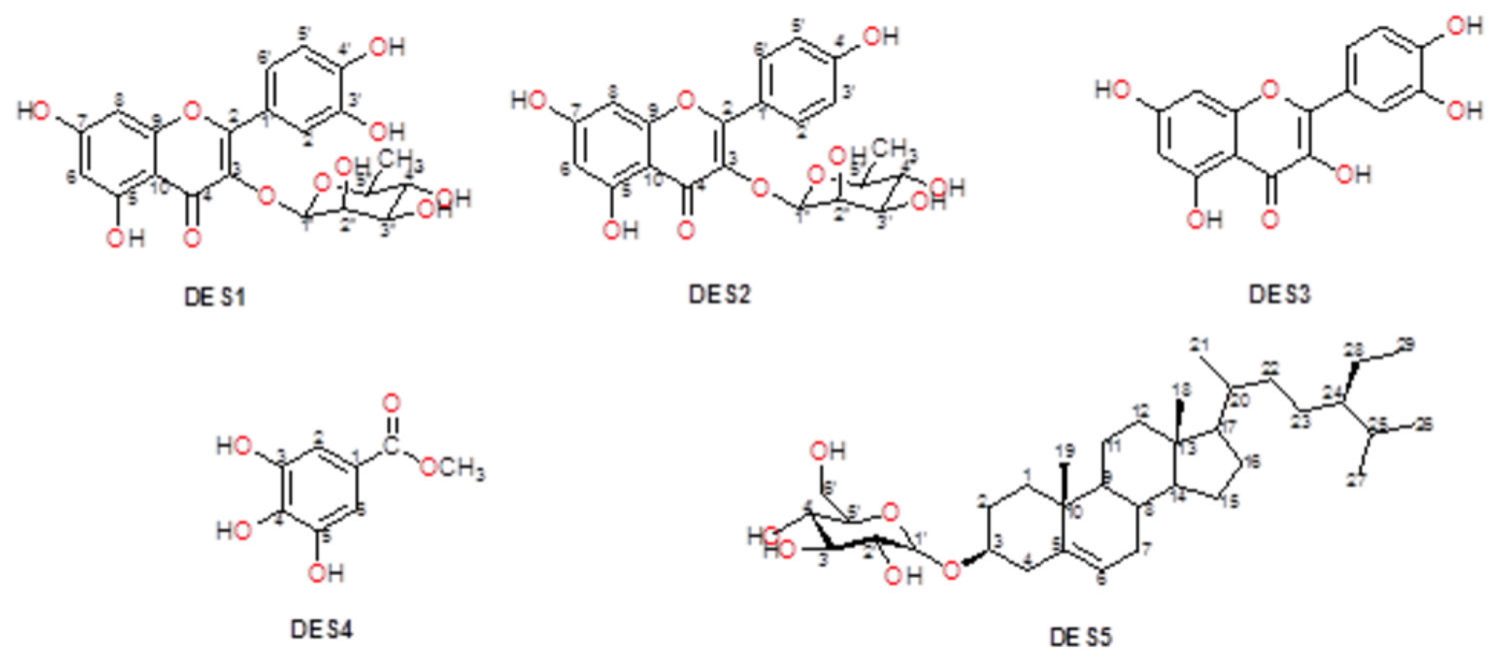
2D structures of the isolated compounds. DES1-Quercitrin, DES2-Afzelin, DES3-Quercetin, DES4-methyl 3,4,5-trihydroxybenzoate ; DES5-Sitosterol 3-O-β-D-glucopyranoside sterol .

Table 1 summarizes the general characteristics of the five compounds isolated from *D. edulis* stem bark. In brief they were all known molecules comprising three flavonoids, one phenolic compound and one sterol. The 2D structures of the different compounds are shown in Figure 1.

**Table 1:** Characteristics of the compounds isolated from the stem bark of *D. edulis*.

**Code**	**Physicochemical properties**	**Extraction yield (mg)**	**Molecular weight (Da)**	Formula and name (reference*)
**DES1**	Yellow powder in CH_2_Cl_2_; Melting point: 176-179 °C, Shinoda test positive	18	~449	C_21_H_20_O_11_; **Quercitrin** [28,29]
**DES2**	Yellow powder in CH_2_Cl_2_; Melting point: 171-173 °C, Shinoda test positive	8	~432	C_21_H_20_O_10_ ; **Afzelin** [29]
**DES3**	Yellow powder in Hex; Melting point: 314-316 °C, Shinoda test positive; reacts with ferric chloride.	10	~302	C_15_H_10_O_7_ ; **Quercetin** [30]
**DES4**	White powder in CH_2_Cl_2_; Melting point: 197-199 °C, reacts with ferric chloride; Shinoda test negative	11	~184	C_8_H_8_O_5_; **methyl 3,4,5-trihydroxybenzoate** [30]
**DES5**	White powder in MeOH; soluble in hot CH_2_Cl_2_ : MeOH (1:1); Melting point: 283-285 °C, Liebermann-Burchard test positive.	8	~579	C_35_H_60_O_6_; **sitosterol 3-O-β-D-glucopyranoside sterol** [31]

* Reference of the work which discovered the compound for the first time.

CH_2_Cl_2_- Methylene chloride; MeOH- Methanol ; Hex- n-hexane ; CH_2_Cl_2_:MeOH- Methylene chloride:Methanol mixture (1:1).

### In vitro antiplasmodial activity

**Figure 2:**
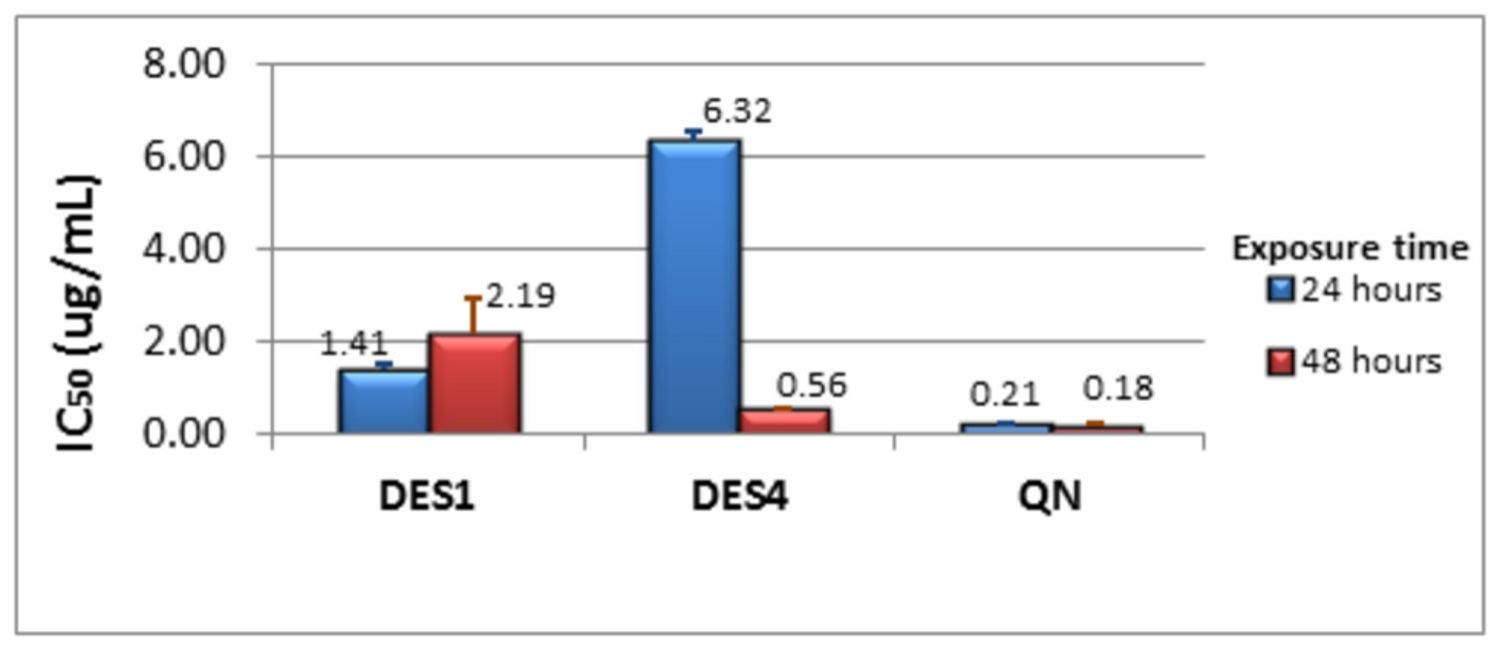
Variation of IC_50_ against Dd2 with exposure time. IC_50_ after 24 hours in blue; IC50 after 48 hours in red. The IC50 values presented were obtained from two distinct experiments run in duplicate each.

The bioactivity profiles of the extracts and isolated compounds are presented in Table 2. The methylene chloride/methanol extract from the stem bark of *D. edulis* showed a significant activity against both Chloroquine-sensitive and resistant strains of *P. falciparum*. The fractionation of this extract yielded five compounds among which DES4 showed the highest activity against both parasite strains (IC_50_ of 0.37 and 0.55 µg/mL respectively). Figure 2 summarizes the variation of the activities of the most active compounds (DES1 & DES4, with IC_50_<5µg/mL against drug-resistant strain) on the parasite growth at different exposure time. DES4 had only limited activity during the first 24 hours, unlike DES1 and Quinine which significantly affected the parasite growth all throughout the life cycle.

**Table 2:** Summary of *in vitro* activity of extracts and isolated compounds.

**Extract / Compound**	**Activity against 3D7**		**Activity against Dd2**		**Cytotoxicity against LLC-MK2**	
	***IC_50_***	***IC_95_***	***IC_50_***	***IC_95_***	***CC_50_***	***Selectivity index/Dd2***
**CH_2_Cl_2_:MeOH**	4.34±0.06	15.04±2.64	6.43±0.89	17.66±6.44	>1000	> 155
**DES1**	5.96±0.51	18.53±3.13	2.26 ±0.28	23.82±6.35	>100	> 44
**DES2**	4.59±0.21	13.64±0.48	19.34±1.56	35.03±3.52	>100	> 5
**DES3**	6.07±0.34	15.19±3.88	5.91±0.97	15.96±4.79	>100	> 17
**DES4**	0.37±0.07	2.58±2.04	0.55±0.06	5.89±0.95	>100	> 182
**DES5**	1.90±0.22	4.38±0.87	5.34±0.98	21.35±0.44	>100	> 18
**ART**	0.02±0.01	0.08±0.02	0.03±0.01	0.12±0.09	ND	ND
**QN**	0.13±0.01	0.48±0.10	0.12±0.01	0.38±0.08	ND	ND

CH_2_Cl_2_:MeOH- Methylene chloride:Methanol (1:1) extract;

DES1-Quercitrin, DES2-Afzelin, DES3-Quercetin, DES4- methyl 3,4,5-trihydroxybenzoate ; DES5-sitosterol 3-O-β-D-glucopyranoside sterol; ART: artemether; QN: Quinine sulphate.

IC_50_, IC_95_ and CC_50_ values are given in µg/mL

The criterion for cytotoxicity used was: CC_50_<1.0 µg/mL (high cytotoxicity); CC_50_ 1.0 -10.0 µg/mL (moderate); CC_50_ 10.0-30.0 µg/mL (mild); and CC_50_ > 30 µg/mL (non-toxic) [22]. The selectivity index defined as SI = CC_50_/IC_50_ was equally considered for compounds with mild cytotoxicity with SI<10 for toxic products [27]. Based on these cut-off values, all products isolated from *D. edulis* in the present study were non-toxic against the LLC-MK2 cell-line.

### In silico “Drug-likeness” profile

**Figure 3:**
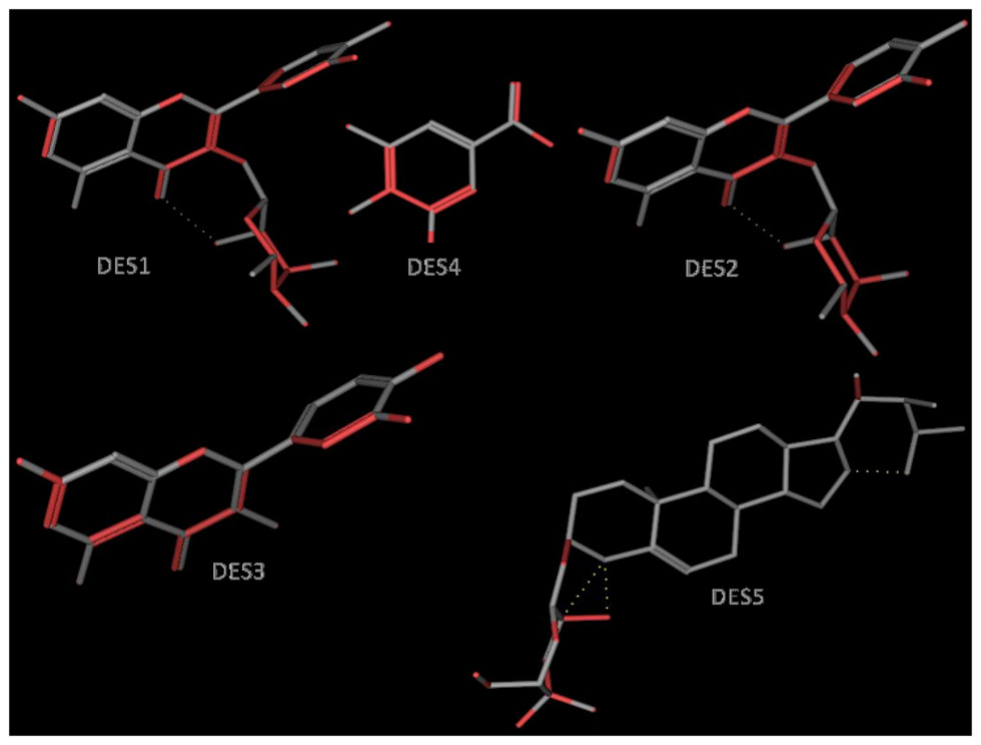
Geometry optimized 3D structures of compounds isolated from *D. edulis*, used to compute molecular descriptors. Carbon atoms are shown in green (DES1 & DES2) and cyan (DES3, DES4 & DES5), while oxygen atoms are shown in red. Hydrogen atoms are left out of the picture for the sake of clarity. The molecular models have been designed using the PyMOL software [47].

The geometry optimized structures are shown in Figure 3, while the computed descriptors are provided in Table 3 (reorder table numbering). Our calculations showed that two of the isolated compounds (DES3 and DES4) have no Lipinski violations (LVs), while DES2 showed only one violation and two compounds (DES1 and DES5) showed two violations. Additionally, DES1 and DES2 had an exceptionally high total polar surface area (TPSA, measured in Å^2^), when compared to the other compounds. The TPSA of DES1 and DES2 were respectively double those of DES5 and DES4. Of the 24 pharmacokinetics-related descriptors, the most potent compound, DES4, showed that all computed descriptors fell within the correct range for > 95% of known drugs (#stars parameter = 0). Compounds DES2 and DES3 also showed compliance, while compounds DES1 and DES5 respectively showed 1 and 4 violations (Table 3).

**Table 3:** Summary of computed *in silico* “drug-likeness” properties of isolated compounds.

**Compound**	**MW (Da)**	***log P*_o/w_**	**HBA**	**HBD**	**NRB**	**TPSA (Å^2^)**	**LV**
**DES1**	448.38	0.803	^*^11	^*^7	3	186.37	2
**DES2**	432.38	1.076	10	^*^6	3	166.14	1
**DES3**	302.24	2.032	7	5	1	127.45	0
**DES4**	184.15	0.993	5	3	2	86.99	0
**DES5**	^*^578.86	^*^6.157	6	4	^**^9	99.38	2

DES1-Quercitrin, DES2-Afzelin, DES3-Quercetin, DES4- methyl 3,4,5-trihydroxybenzoate ; DES5-3-O-β-D-glucopyranosyl sitosterol

* Violations of Lipinski’s “Rule of Five” (ro5); ^**^ Violations of additional rule for NRB.

### Drug-interaction studies

**Figure 4:**
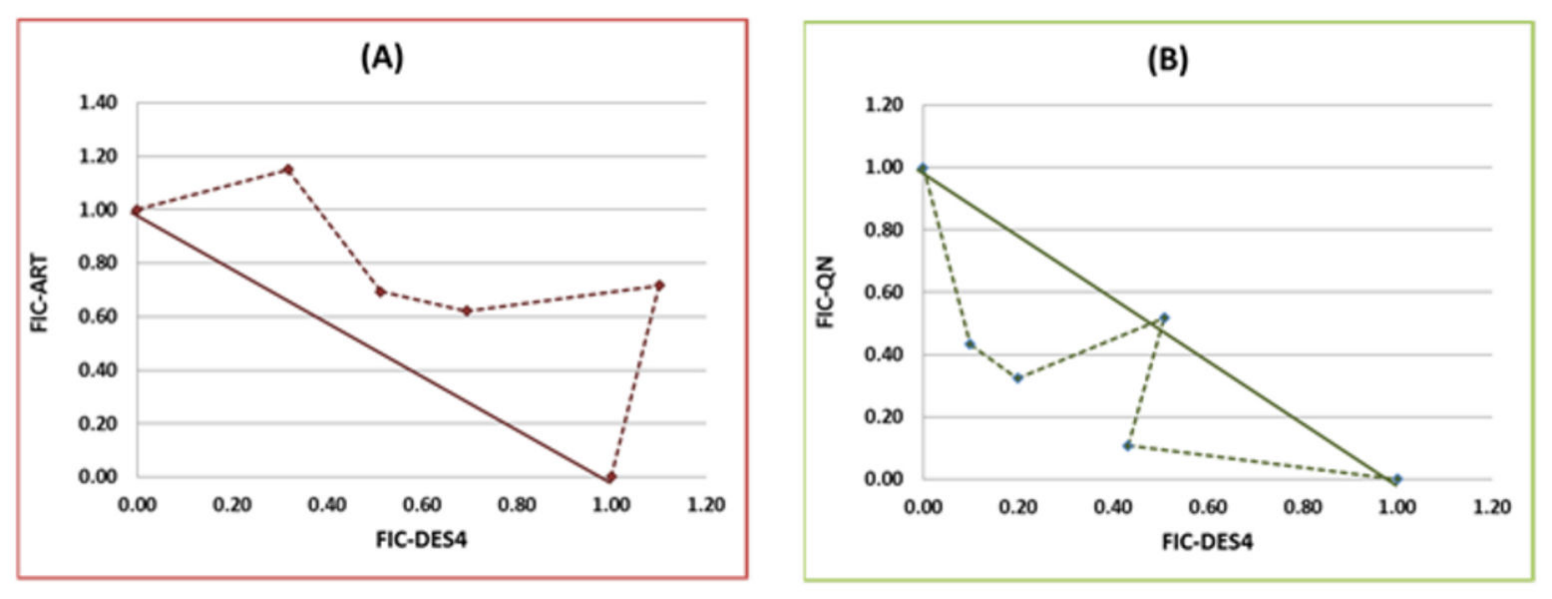
Isobolograms showing interactions of DES4 with Artemetherand Quinine, respectively. (**A**): DES4 *vs* Artemether (ART); (**B**): DES4 *vs* Quinine (QN).

Drug-interactions of DES4 with artemether and quinine respectively, are summarized in Figure 4. Combination Index between DES4 and artemether varied from 1.05 to 1.19 with an overall value of 1.10, indicating additivity between the two drugs. However, the graphical analysis showed a neat trend toward antagonism between the two drugs, illustrated by a concave curve (Figure 4A). For quinine all the combinations showed ∑FIC < 1, except for Combination 2 (CI=1.05), indicating a synergistic effect of DES4 – ART combination against Dd2; CI ranged from 0.52 to 1.03, with a mean value of 0.65 which is a clear indication of synergistic interaction between the two drugs. The isobologram (Figure 4B) showed a convex plot confirming the synergistic effects of the two drugs with regards to each other. Combination 4 (DES4:QN, 2:3) showed the lowest CI (0.52). With this formula, the IC50s of DES4 and QN were 310 and 31 ng/mL respectively. Considering both drugs in combination as a whole, the IC50 obtained was 330 ng/mL. 

## Discussion

The present investigation is the first to demonstrate significant antiplasmodial activities in pure compounds isolated from the stem back of a widely used food and medicinal plant *Dacryodes edulis*. 

From its spectral data, DES1 was identified to be Quercitrin or Quercetin-3-*O-α-L*-rhamnopyranoside, a compound commonly found in plants, especially in glycosylated form [28,29]. Spectral data on DES2 revealed this compound as Afzelin, a 3’-dehydroxylated form of Quercitrin. This molecule has equally been isolated from *Embelia concinna* [30]. The structure of compound 3 was found to be perfectly similar to a portion of Quercitrin. But, considering the quantity obtained (10 mg against 18 mg of Quercitrin), the hypothesis of this compound occurring only as a result of cleavage of quercitrin is hardly plausible. Moreover, literature search revealed compound 3 as a well-known molecule, quercetin (deglycosyl-quercitrin) earlier identified by analytical HPLC-MS isolated from the fruits extract of *D. edulis* by Atawodi et al. [31]. The fruits of *D. edulis* carried out by these authors equally led to the identification of methyl 3,4,5-trihydroxybenzoate which was also isolated from the stem bark in the present study, and codified as DES4. DES5, sitosterol 3-O-β-D-glucopyranoside, was previously isolated by Ramiarantsoa et al. [32] from the leaves of *Ravanda madagascariensis.*

The antiplasmodial activities of the isolated compounds ranged from 0.37 to 6.07 μg/mL against 3D7, and 0.55 to 19.34 μg/mL against the multidrug-resistant Dd2 strain. The most active of the five compounds identified (Table 1, Figures 1 & 3) was DES4 which was earlier isolated, but not tested for antiplasmodial activity. Three of the four remaining molecules showed moderate activity, with IC_50_ below 10 μg/mL, against both parasite strains. These compounds are Quercitrin, Sitosterol 3-O-β-D-glucopyranoside and Quercetin, while Afzelin was inactive against the multidrug-resistant Dd2. More interestingly, all the tested extracts and compounds were found to be non-toxic (CC_50_>30 µg/mL) against the LLC-MK2 monkey kidney epithelial cells. This observation may be an indicator of their lack of serious toxicity in mammalian organisms. In fact, *D. edulis* is widely used in many African countries including Nigeria and Cameroon, both for therapeutic and nutritional purposes [33]. No part of *D. edulis* is known to be toxic, except for the presence of some anti-nutrient factors such as oxalate, tannins, Phytates and Trypsin-inhibitors in the seeds [3,34]. 

The present study therefore is a step towards identifying some of the molecules responsible of the antimalarial exhibited by this plant. Preliminary investigation of the stage-specific activity of the most active compounds, DES4 and DES1 was also carried out, and showed that DES4 targets the late stages of the erythrocytic cycle (late trophozoite and schizont) preferably, unlike DES1 whose inhibitory effects were observed on both the early and late stages of the erythrocytic cycle. However, if this assay has a merit of detecting drugs targeting early parasite stages like rings and young trophozoites, is not complete enough to rationally confirm the mechanism of action specific to late-stage parasite. The drop in viability and/or multiplication observed later may also be due to a slow action or accumulation of the drug in the parasite. Further investigations are needed to rationally identify the target(s) in the malaria parasite, as well as to elucidate its mechanism of action, in order to optimize the anti-malarial potential of the molecule.

Lipinski’s “Rule of Five” (ro5) is often considered as a useful test for the evaluation of oral availability for compounds in the early stages of drug discovery protocols [35]. Even though natural products (NPs) of plant origin often fail the famous Lipinski “drug-likeness” test [36], they are often rich in stereogenic centres and cover segments of chemical space which are typically not occupied by a majority of synthetic molecules and drugs [36,37]. In summary, Lipinski’s ro5 defines an orally available molecule as one for which the molar weight (MW) ≤ 500 Daltons (Da), the logarithm of the *n*-octanol/water partition coefficient [log P_(o/w)_] ≤ 5, the number of hydrogen bond acceptors (HBA) ≤ 10 and the number of hydrogen bond donors (HBD) ≤ 5. An additional rule for the number of rotatable bonds (NRB) is often added to this ro5, such that NRB ≤ 5. Additionally, the structural and hence physico-chemical properties of isolated phytochemicals are often fine-tuned by chemical synthesis, leading to “drug-like” molecules with desirable ADME/T (absorption, distribution, metabolism, excretion, and toxicology) properties. These are often referred to as natural product inspired drugs [39,40]. Thus, modern drug discovery programs often resort to natural sources to guide the careful design of “drug-like” leads from suitable scaffolds, often by synthetic modifications of the latter [38-41]. In this study, we have evaluated the “drug-likeness” of the compounds isolated from *D. edulis* by using Lipinski criteria, from computed molecular properties of the geometry optimized structures of the NPs. An evaluation of “lead-likeness”, with more stringent criteria (150 ≤ MW ≤ 350; log P_(o/w)_ ≤ 4; HBD ≤ 3; HBA ≤ 6) [42-44], and “fragment-likeness” (MW ≤ 250; -2 ≤ log P_(o/w)_ ≤ 3; HBD < 3; HBA < 6; NRB < 3) [45], demonstrated that only DES4 could be further developed into a lead compound for drug discovery against malaria. Additionally, it is important to note that the most active compound (DES4) showed Lipinski compliance, and also passed the “lead-likeness” and “fragment-likeness” tests, qualifying it as a potential candidate for antimalarial drug discovery. DES4 has a unique scaffold, unlike the flavonoids (DES1, DES2 and DES3) and the steroid (DES5), implying that the size is an important factor to consider when designing more active analogues of the active DES4. This further suggests that the receptor site to which the active compound (ligand) binds should be a pocket of restricted size, to which the larger (less active ligands) do not conveniently fit. The –OH groups at positions 3, 4 and 5 (Figure 1) could also play key roles as donors, since hydrogen bond formation at the receptor site appears to be the key binding interaction in ligand-receptor binding for this active DES4 Substitutions at positions 1, 3, 4 and 5 on the benzene ring could lead to the generation of a combinatorial library in the ligand optimization stage of drug discovery, suggesting that DES4 could be a good starting point for lead discovery against malaria. Many molecules and drugs fail at later stages of drug discovery because of poor absorption, distribution, metabolism, excretion and toxicity (ADMET) properties [46]. It has thus become imperative to predict such properties of drugs at early stages of drug discovery protocols using *in silico* methods. In this study, we have computed 24 most relevant ADMET-related molecular descriptors for each of the isolated compounds and used as predictors for assessing their drug metabolism and pharmacokinetic (DMPK) properties. If all predicted properties of the isolated molecule fall within the acceptable range for 95% of known drugs, a compliance score (#stars = 0) is attributed. Otherwise, #stars = *x*, where *x* represents the number of times the computed descriptors fall outside the given range. In addition to the ro5 (Table 3), a summary of 8 computed descriptors for all five isolated compounds is shown in Table 4. For DES2, DES3 and DES4, all 24 relevant descriptors related to DMPK fall within the acceptable range for 95% of known drugs. The geometry optimized 3D structures of the different compounds, designed using PyMOL software [47] are shown in Figure 3.

**Table 4:** Summary of some computed *in silico* pharmacokinetics properties of isolated compounds.

^a^Cpd	^*b*^#stars	^c^ro3	*^d^_log Swat_*	^*e*^ *BIP *_caco−2_(*nm s* ^−1^)	*^f^_log KHSA_*	^g^MDCK	^h^logB/B	^i^logHerg	^*j*^#metab
**DES1**	1	2	-3.03	9.82	-0.61	3.34	-3.00	*-5.18	7
**DES2**	0	0	-3.21	25.38	-0.51	9.33	-2.52	*-5.30	6
**DES3**	0	1	-2.76	22.45	-0.35	8.17	-2.27	-4.95	5
**DES4**	0	0	-1.32	^*^130.79	-0.67	54.89	-1.33	-3.65	3
**DES5**	4	1	^*^-7.65	^*^230.95	0.98	101.48	-2.20	*-5.55	6

DES1-Quercitrin, DES2-Afzelin, DES3-Quercetin, DES4- methyl 3,4,5-trihydroxybenzoate ; DES5-3-O-β-D-glucopyranosyl sitosterol

* Compound property falling outside the range for 95% of known drugs; ^*a*^Compound isolated from *D. edulis*; ^*b*^Number of properties falling outside the range for 95% of known drugs; ^*c*^Number of violations of Jorgensen’s “Rule of Three” (*log S*_*wat*_> -5.7, *BIP *_*caco*−2_> 22 nm/s and # Primary Metabolites < 7); ^*d*^Logarithm of aqueous solubility (range for 95% of drugs: - 6.0 to 0.5); ^*e*^Predicted apparent Caco-2 cell membrane permeability in Boehringer Ingelheim scale, in nm/s (range for 95% of drugs: < 5 low, > 100 high); ^*f*^Logarithm of predicted binding constant to human serum albumin (range for 95% of drugs: - 1.5 to 1.2); ^*g*^Predicted apparent Madin-Darby canine kidney (MDCK) cell permeability in nm/sec (< 25 poor, > 500 great); ^*h*^Logarithm of predicted blood/brain barrier partition coefficient (range for 95% of drugs: - 3.0 to 1.0); ^*i*^Predicted logarithm of IC_50_ value for blockage of HERG K^+^ channels (concern < -5); ^*j*^Number of likely metabolic reactions (range for 95% of drugs: 1–8).

Equally investigated was the drug-interaction patterns of DES4 with Artemether and Quinine. Combining DES4 and quinine increased their activities by 3 and 6-fold respectively, revealing synergistic interactions between the two compounds. In contrast, only additive effects were observed between DES4 and artemether. The present study showed that DES4 is likely to serve as lead in the development of partner drugs in quinine-based combination therapies. Further studies including *in vivo* and toxicological testing of the combinations are essential to step forward in the exploration of these ground findings. 

## Conclusion

The findings from this work could serve as basis for further investigations towards developing new antimalarial leads from *D. edulis* compounds, especially DES4 (methyl 3,4,5-trihydroxybenzoate). This compound was Lipinski compliant and also respected the tests for “lead-likeness” and “fragment-likeness”. In addition, all computed descriptors related to drug metabolism and pharmacokinetics (DMPK) point to the fact that these properties fall within the acceptable ranges for 95% of known drugs. A pharmacophore-based method could be used to virtually screen for potential hits with similar pharmacophore features as DES4, from suitable database like the recently developed CamMedNP library [48]. 
